# Genome-wide analysis of lncRNA and mRNA expression and endogenous hormone regulation during tension wood formation in *Catalpa bungei*

**DOI:** 10.1186/s12864-020-07044-5

**Published:** 2020-09-05

**Authors:** Yao Xiao, Fei Yi, Juanjuan Ling, Guijuan Yang, Na Lu, Zirui Jia, Junchen Wang, Kun Zhao, Junhui Wang, Wenjun Ma

**Affiliations:** 1 State Key Laboratory of Tree Genetics and Breeding, Key Laboratory of Tree Breeding and Cultivation of State Forestry and Grassland Administration, Research Institute of Forestry, Chinese Academy of Forestry, Beijing, 100091 PR China; 2 Luoyang Academy of Agriculture and Forestry Science, Luoyang, 471002 Henan Province China

**Keywords:** Tension wood, *Catalpa bungei*, Phytohormones, lncRNA, Regulatory network

## Abstract

**Background:**

Phytohormones are the key factors regulating vascular development in plants, and they are also involved in tension wood (TW) formation. Although the theory of hormone distribution in TW formation is widely supported, the effects of endogenous hormones on TW formation have not yet been assessed. In this study, TW formation was induced in *Catalpa bungei* by artificial bending. The phytohormone content of TW, opposite wood (OW) and normal wood (NW) was determined using liquid chromatography-mass spectrometry (LC-MS), and transcriptome sequencing was performed. The hormone content and related gene expression data were comprehensively analyzed.

**Results:**

The results of analyses of the plant hormone contents indicated significantly higher levels of cis-zeatin (cZ), indoleacetic acid (IAA) and abscisic acid (ABA) in TW than in OW. Genes involved in the IAA and ABA synthesis pathways, such as *ALDH* (evm.model.group5.1511) and *UGT* (evm.model.scaffold36.20), were significantly upregulated in TW. and the expression levels of *ARF* (evm.model.group5.1332), *A-ARR* (evm.model.group0.1600), and *TCH4* (evm.model.group2.745), which participate in IAA, cZ and Brassinolide (BR) signal transduction, were significantly increased in TW. In particular, *ARF* expression may be regulated by long noncoding RNAs (lncRNAs) and the HD-ZIP transcription factor ATHB-15.

**Conclusions:**

We constructed a multiple hormone-mediated network of *C. bungei* TW formation based on hormone levels and transcriptional expression profiles were identified during TW formation.

## Background

Trees have highly lignified stems, which are the source of wood. Wood is the main material used for production and homebuilding. Strategies for improving wood yields and quality have historically been the focus of tree breeders. The discovery of reaction wood provided a new approach for use by researchers to study the mechanism of wood formation [1, 2]. Reaction wood is classified into tension wood (TW) and compression wood according to the orientation of the stress in the xylem (angiosperms generate stronger tension stresses on the upper side of the stem, and gymnosperms generate stronger compression stresses on the lower side of the stem) [3]. TW usually exists in asymmetric xylem and exhibits extremely high growth stress levels. Trees regulate the stem growth direction through eccentric growth [4]. The physical and chemical properties of TW are very different from normal wood (NW). Mechanically, TW has an unusually high tensile strength and dry shrinkage, but its compressive strength is very low [5, 6]. Regarding its chemical composition, studies have reported a low lignin content and high arabinogalactan protein content in TW [7, 8]. The processing of wood containing TW is fraught with problems, such as cracking. The processing of wood containing TW is fraught with problems such as cracking restricting its use as high-quality timber. However, TW provides the basis for exploring the processes of wood development that result in unique physical and chemical properties. Therefore, studies examining the mechanism of TW formation are very important.

The mechanism of TW formation was studied as early as the middle of the twentieth century, but the cause of TW formation has not been assessed. Researchers have formulated (1) growth stress hypotheses [9], (2) the gravitational response theory [10], and (3) the auxin distribution theory to explain TW formation. The auxin distribution theory has been accepted by many scholars, and its principle is that the distribution of endogenous hormones is not balanced between the upper and lower stems due to the effects of gravity when tree growth is not vertical. This imbalance leads to cell division in the upper or lower stem, widening of the growth ring, and the production of specialized cell structure, resulting in TW formation [11, 12]. The anatomy of TW is vastly different from NW. Generally, TW is described as having small vessels and a high ratio of fibers to vessels and a gelatinous layer (G-layer) is included in the fiber lumen in some tree species [13, 14]. The specialization of these cells and tissues is often regulated by endogenous hormones. According to Love et al. (2009), characteristics of xylem treated with ethylene are similar to the characteristics of TW, both of which generate eccentric growth and decrease the proportion of vessels [15]. Felten et al. (2018) compared the xylem development of wild-type and ethylene-insensitive hybrid poplars following ethylene application and found that ethylene activated some transcription factors and triggered G-layer formation [16]. Moreover, ethylene was recently shown to be a key phytohormone regulating TW formation [14]. Gibberellin (GA) enhances the activity of cambium [17], and xylem specialization may be caused by changes in vascular cambium function. Gibberellin has consistently been shown to promote TW formation [18, 19]. Part of the regulatory mechanism has also been explained as follows: gibberellin induces the degradation of the PtRGA protein, alleviates the inhibition of PtFLA by PtRGA, promotes PtFLA gene expression and eventually induces poplar TW formation [20]. However, other studies have not reported a significant correlation between gibberellin and G-layer differentiation [21]. In addition, exogenous indoleacetic acid (IAA) exerts a significant regulatory effect on xylem formation. After IAA treatment, the secondary xylem of hybrid poplar forms TW, with a concomitant increase in the expression of the genes related to auxin transport and cellulose biosynthesis [22]. As shown in the study by Hellgren et al. (2004), the endogenous IAA content in TW is not significantly different from that in NW but was higher than in OW [23]. However, some studies did not reach this conclusion; instead, a significant difference in the endogenous IAA content was not observed between TW and OW and the authors proposed that the change in the IAA distribution did not cause TW formation [24]. Researchers have not clearly determined whether the effect of endogenous IAA on TW formation is positive or negative [3]. Most studies focus on the regulation of plant growth by a single hormone but rarely discuss the regulation of growth mediated by interactions among hormones. On the other hand, a large number of studies have also explored gene expression during TW formation. Genes related to cell wall formation expressed in TW, such as cellulose synthase and pectin metabolism genes, displayed significant differences from genes expressed in NW or OW [25–27]. Gerttula et al. (2015) generated genome-wide transcriptomes for trees in which gene expression was perturbed by gravistimulation, GA treatment, and modulation of *ARK2* expression, and the transcription factor ARK2 altered TW formation in poplar by regulating gibberellin signaling [28]. In addition to transcription factors, long noncoding RNAs (lncRNAs) are also involved in TW formation as transcriptional regulators. Chen et al. (2014) conducted the genome-scale identification and characterization of lncRNAs in TW of *Populus* and the authors documented that predicted target genes of lncRNAs are involved in many biological processes, such as cellulose, lignin and gibberellin biosynthesis, suggesting potential roles of lncRNAs in wood formation [29]. Although plant hormones are key factors regulating TW formation, the underlying expression pattern has not been elucidated, particularly the lncRNA expression pattern. The identification of this pattern is one of the aims of our study.

*Catalpa. bungei* has a straight stem with a high wood density, high hardness and strong bending resistance and is considered a valuable commercial tree species [30]. An analysis of the wood formation mechanism of *C. bungei* will facilitate the genetic improvement of its timber. The existence of TW makes the wood prone to distortion and cracking during wood processing. These distortions also substantially hinder the use of precious timber. Therefore, studies examining the pattern of TW formation under physiological conditions and the associated molecular mechanisms are of great practical value for reducing the loss caused by defects due to TW and improving the quality and utilization of wood. Plant growth traits are often controlled by multiple hormones and analyses of the actions of multiple hormones may be a reliable approach for determining the underlying mechanisms. Thus, in the present study, we induced TW formation in *C. bungei* by artificial bending using a clone with high wood density and hardness (Additional file 1). The endogenous hormone levels and the transcriptomes of TW, opposite wood (OW) and NW were investigated to reveal the potential mechanisms by which multiple hormones regulate TW formation via hormone synthesis and signal transduction.

## Results

### The development of fibers and vessels in the TW of *C. bungei*

**Fig. 1:**
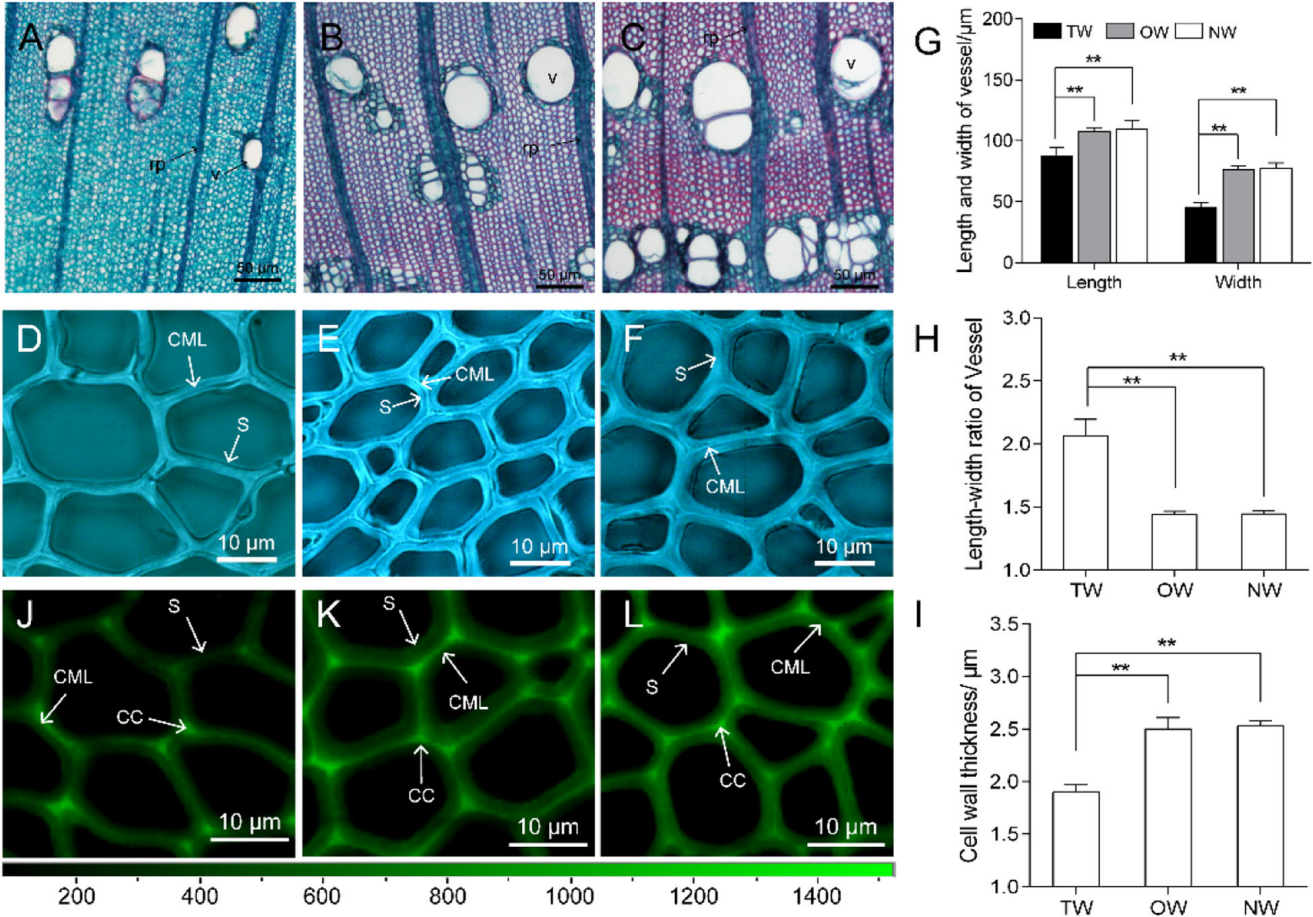
Vessel and fiber cell characteristics of TW, OW and NW. Tension wood (**a**, **d** and **j**). Opposite wood (**b**, **e** and **k**). Normal wood (**c**, **f** and **l**). Vessel (v). Ray parenchyma (rp). Cell corner (CC). Secondary cell wall (S). Cell middle lamella (CML). Second secondary cell wall (S2). ** indicates significant differences at a significance level of 5%

After artificial bending, the xylem development in the upper bent stem in *C. bungei* was significantly altered. The vessel size was smaller and the vessel length and width were significantly smaller in TW than in the OW and NW. However, in TW, the vessel length:width ratio was significantly larger than in OW and NW by approximately 1.4-fold, and the vessels had a long oval shape, which was in contrast to the round vessels in OW and NW (Fig. 1a-c, g and h). The fiber cell lumen of *C. bungei* TW did not contain a G-layer. In addition, the secondary wall thickening was blocked, leading to a thinner secondary wall (Fig. 1i). Moreover, the Raman spectroscopy and microscopy imaging results revealed reduced lignification of the TW fiber cells (Fig 1j-l).

### Content of endogenous hormones in TW, OW and NW

**Fig. 2:**
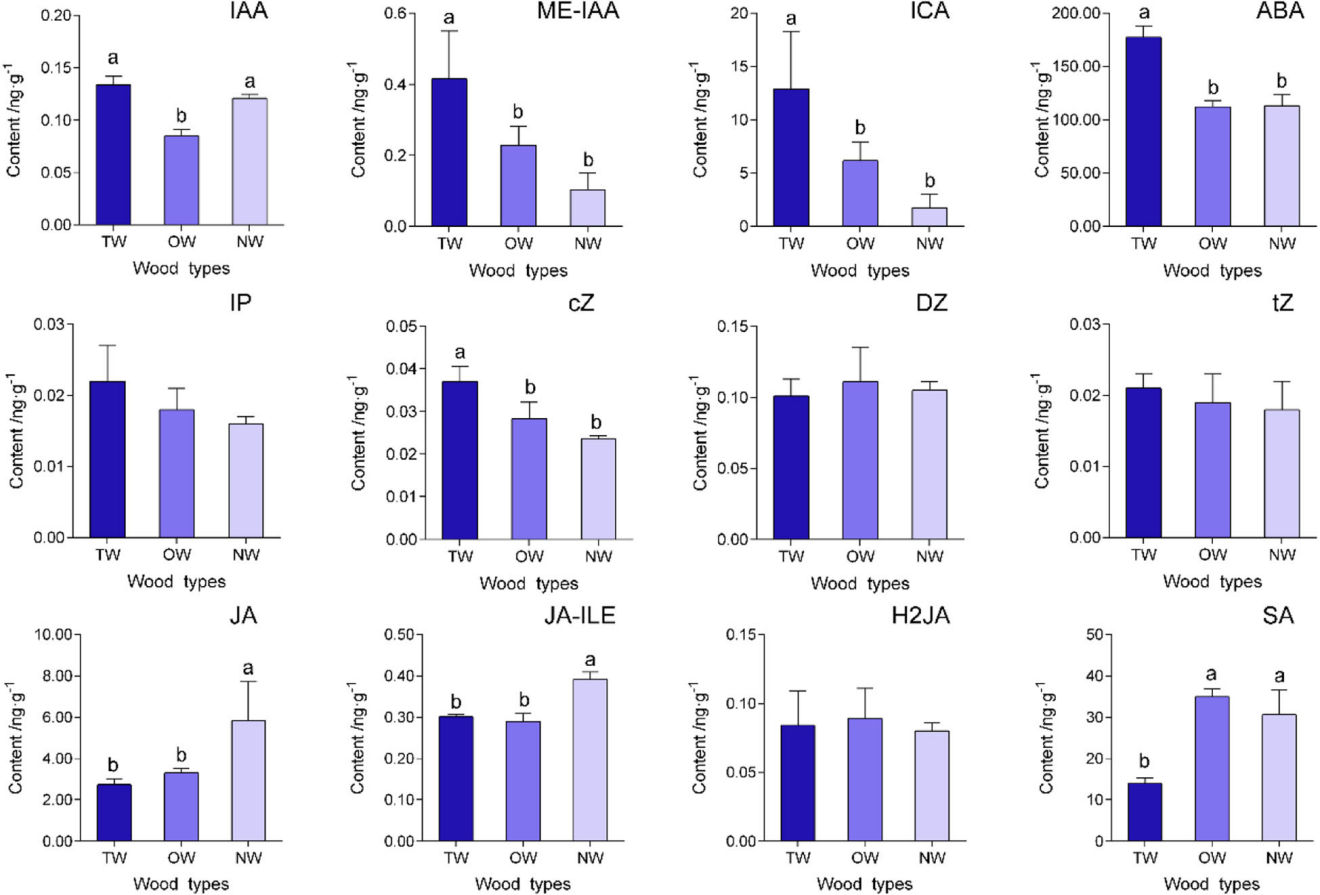
Phytohormone levels in different wood types. The different letters indicate significant differences at a significance level of 5%. IAA: indoleacetic acid, ME-IAA: methyl indol-3-ylacetate, ICA: indole-3-carboxylic acid, ABA: abscisic acid, IP: N6-(Δ2-isopentenyl) adenine, cZ: cis-zeatin, DZ: dihydrozeatin, tZ: trans-zeatin, JA: jasmonic acid, JA-ILE: jasmonoyl-isoleucine, H2JA: dihydrojasmonic Acid, SA: salicylic acid

**Fig. 3:**
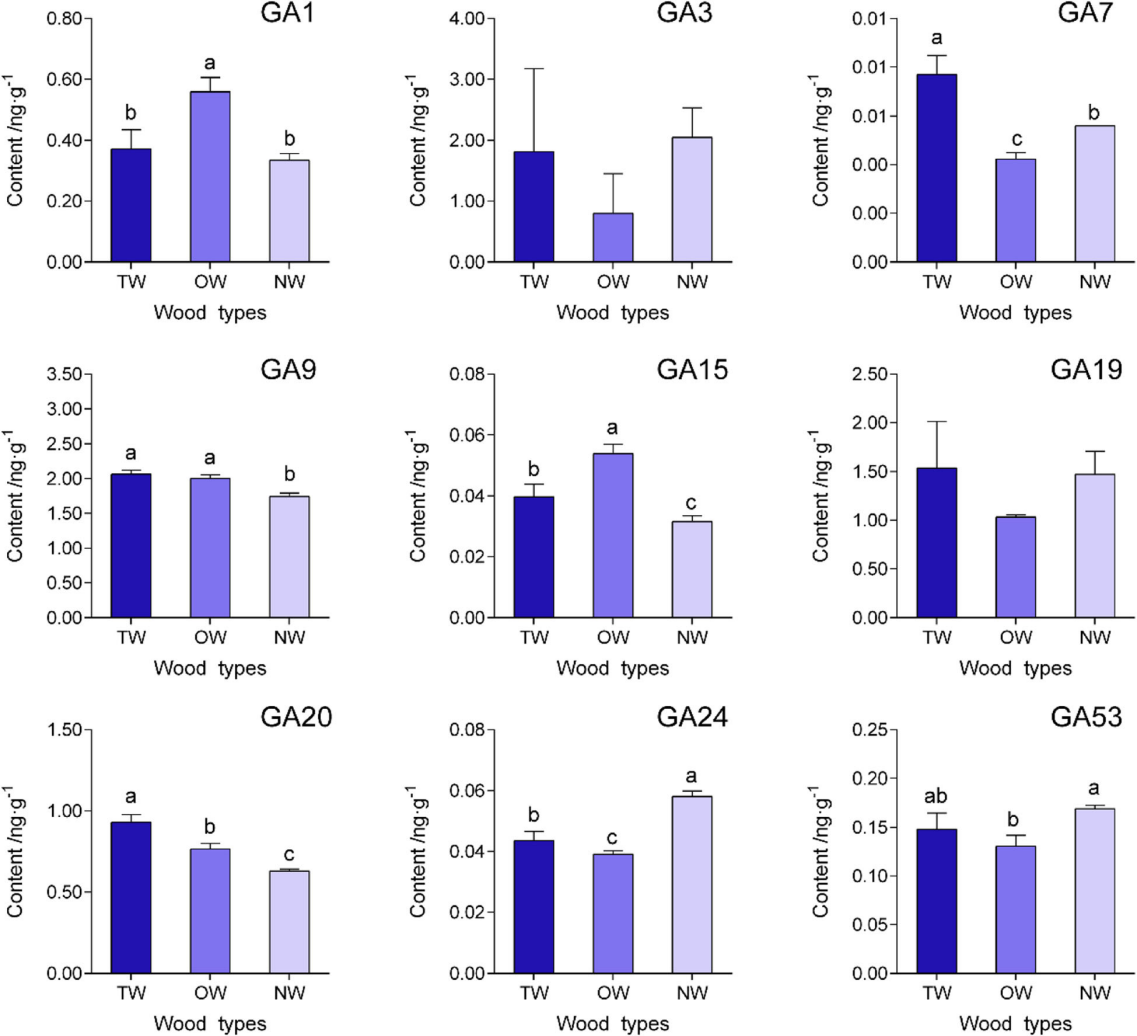
The gibberellin levels in different wood types. The different letters indicate significant differences at a significance level of 5%

In this study, the contents of six major plant hormones—auxin, zeatin, abscisic acid, jasmonic acid, salicylic acid and gibberellin— were determined using liquid chromatography-mass spectrometry (LC-MS). The statistical analysis revealed approximately 58.24% higher contents of indoleacetic acid (IAA), methyl indol-3-ylacetate (ME-IAA) and indole-3-carboxylic acid (ICA) in TW than in OW, but the concentrations did not significantly differ from those in NW (Fig. 2). In addition, the cis-zeatin (cZ) content in TW was significantly higher than in OW and NW. Furthermore, a significantly higher abscisic acid (ABA) level was detected in TW than in OW and NW, and significantly higher JA and SA levels were detected in NW than in TW and OW. Here, a significantly higher GA1 level was observed in OW than in TW and NW, and the highest GA7 level was detected in TW, while the GA3 level did not differ significantly among the three wood types (Fig. 3).

### Differential expression of mRNAs and lncRNAs

**Fig. 4:**
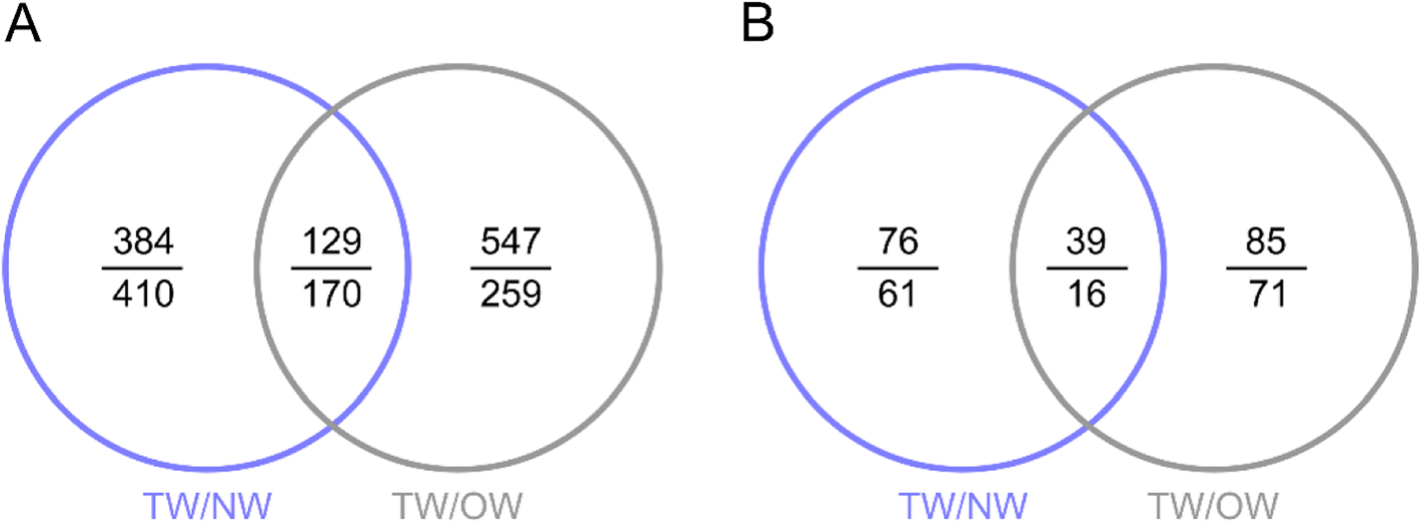
Numbers of differentially expressed transcripts in different comparison groups: mRNAs (**a**), lncRNAs (**b**). The number of upregulated genes is above the line, the number of downregulated genes is under the line

We measured the transcript abundance in TW, OW and NW to further analyze the internal regulatory mechanism of TW formation. A total of 8998 novel lncRNAs were detected. Intergenic lncRNAs were the most abundant type, Overall, 554 antisense lncRNAs were detected, but no intronic lncRNAs were identified (Additional file 2). According to the transcript expression analysis (Fig. 4), 384 and 410 mRNAs were upregulated and downregulated only in TW/NW, respectively. There were Five hundred forty-seven mRNAs significantly upregulated and 259 mRNAs were significantly downregulated in TW compared with OW. One hundred fifteen and 124 lncRNAs were significantly upregulated in TW compared with NW and OW, respectively. Seventy-seven and 87 lncRNAs were significantly downregulated in TW/NW and TW/OW, respectively. Thirty-nine of these lncRNAs were upregulated in two comparison groups and 16 lncRNAs were downregulated in two comparison groups.

### Analysis of differentially expressed genes (DEGs) involved in plant hormone biosynthesis and signal transduction pathways

**Fig. 5:**
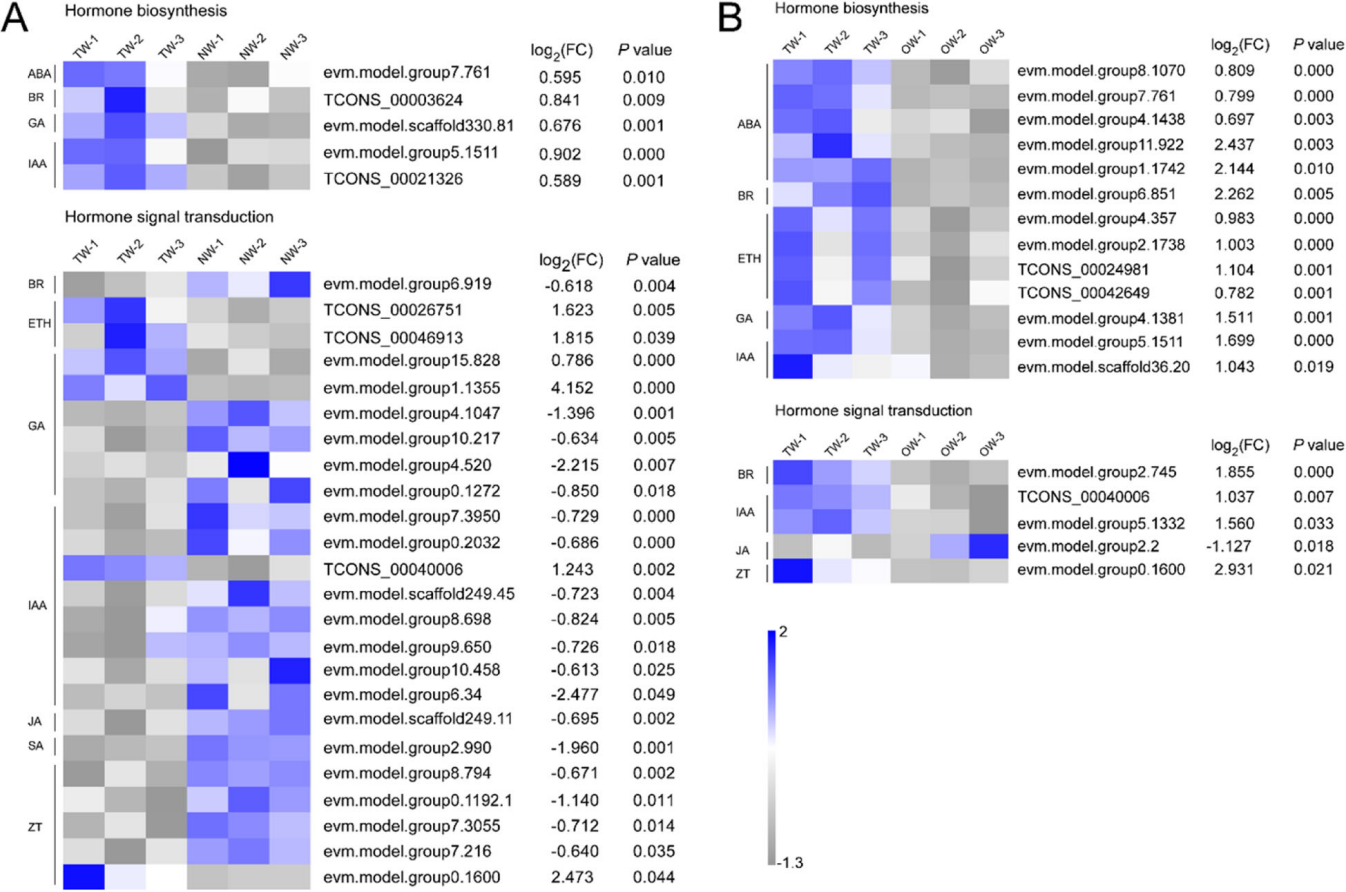
Expression levels of transcripts involved in phytohormone synthesis and signal transduction pathways in the TW/NW (**a**) and TW/OW (**b**) comparison groups. (FC), fold change value

According to the Kyoto Encyclopedia of Genes and Genomes (KEGG) database and targeted analysis of the plant hormone synthesis pathway and signal transduction pathway, the DEGs in the TW/NW and TW/OW comparison groups (Fig. 5a-b) with an expression level threshold of (FPKM) > 1, fold change > 1.5 or < − 1.5 and a *P* value < 0.05 were selected (Additional file 3). In these two comparison groups, 5 and 13 DEGs related to plant hormone biosynthesis and catabolism were significantly upregulated in the TW/NW and TW/OW comparison groups, respectively. Additionally, more genes involved in plant hormone signal transduction were significantly differentially expressed between TW and NW. (Additional file 3).

**Fig. 6:**
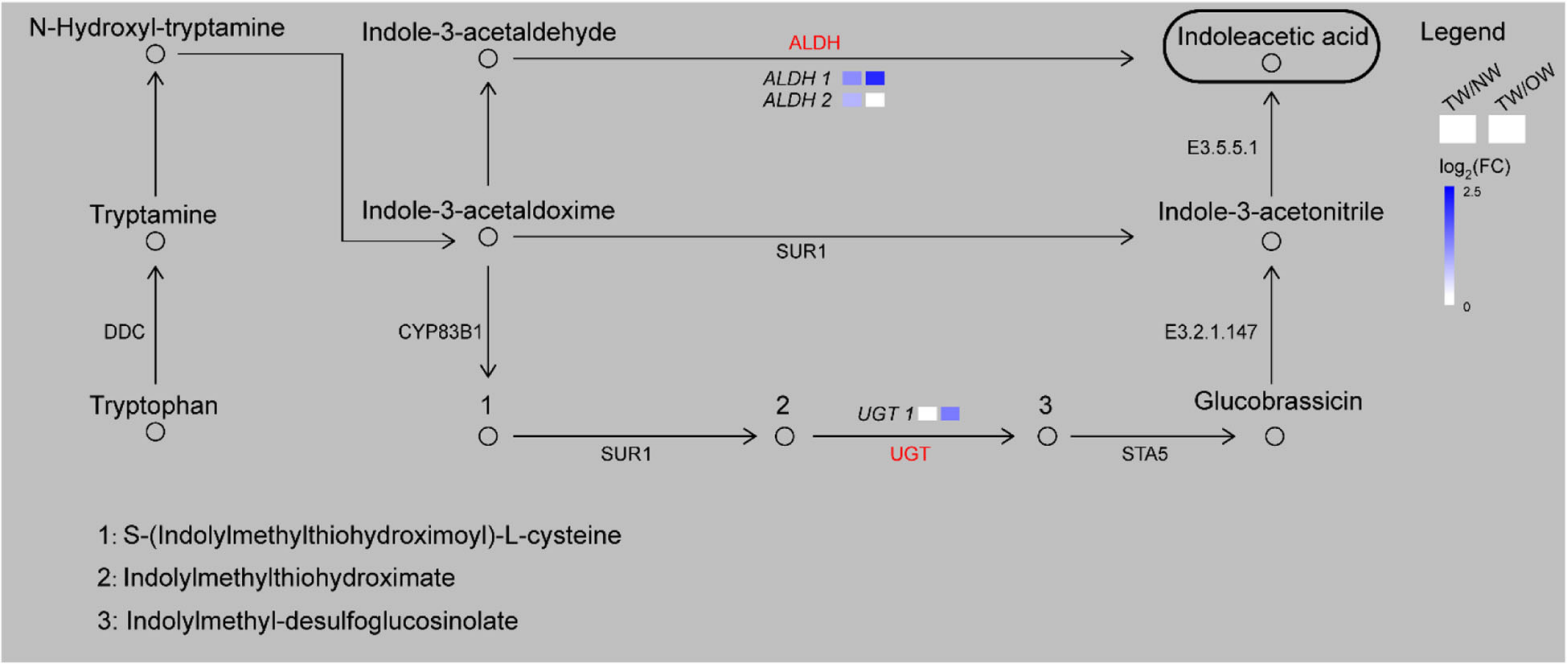
The differentially expressed transcripts involved in auxin synthesis. (FC), fold change value

**Fig. 7:**
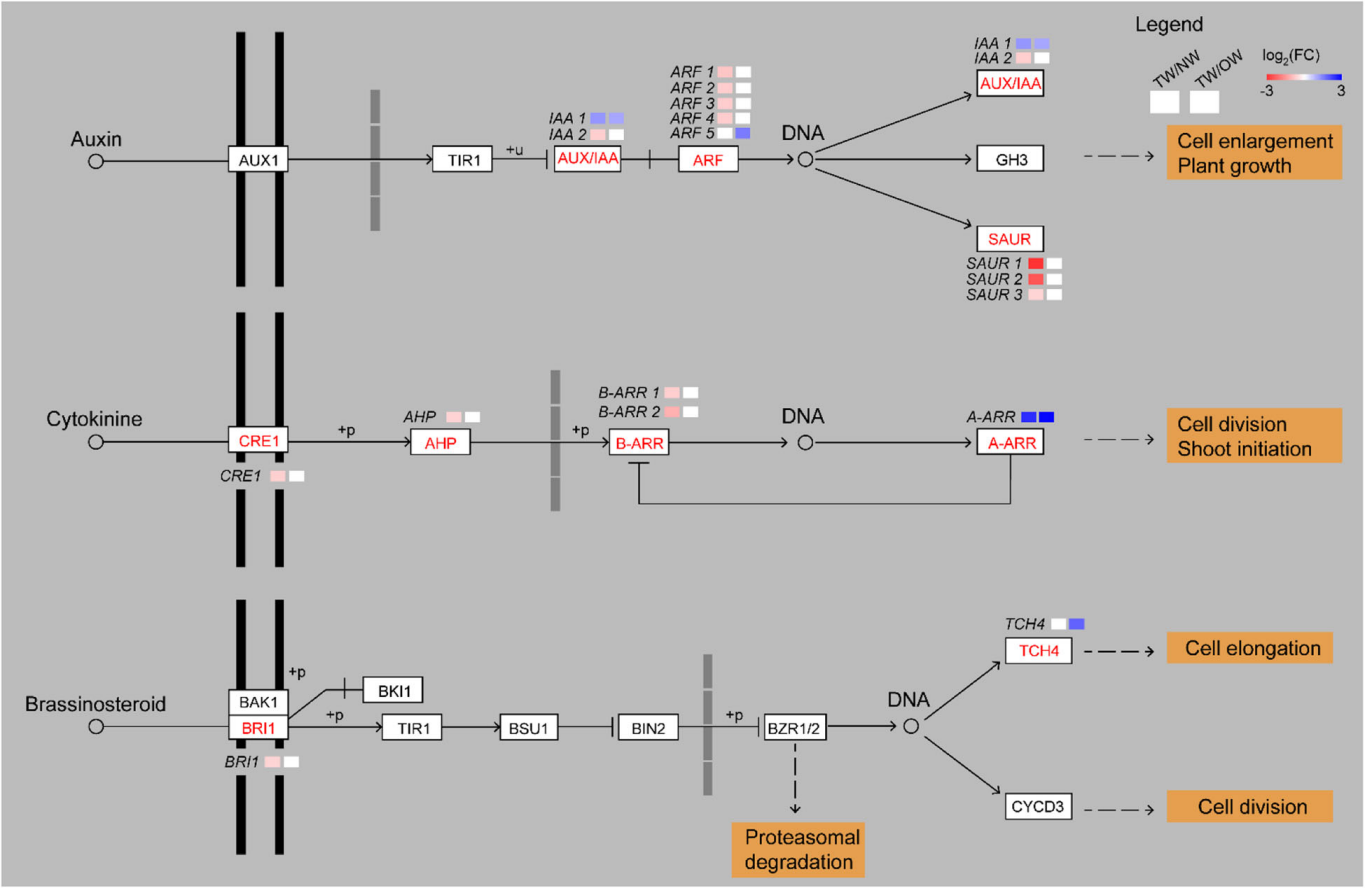
The differentially expressed transcripts involved in the plant hormone signal transduction pathway. (FC), fold change value

In this study, a significantly higher cZ level was detected in TW than in OW and NW, and the genes responding to zeatin were also significantly differentially expressed (Figs. 2; 5A-B and 7). *A-ARR* (evm.model.group0.1600) transcript was significantly upregulated in TW compared with both NW and OW. The *CRE*, *AHP* and *B-ARR* genes were downregulated in TW compared with NW. On the other hand, the BR receptor gene *BRI1* (evm.model.group6.919) was significantly downregulated in TW compared with NW. However, in the TW/OW comparison, *CYP90B1* (evm.model.group6.851), which catalyzes the synthesis of BR, and the *TCH4* transcript (evm.model.group2.745), which responds to BR, were significantly upregulated. (Figs. 5b and 7). In addition, because the IAA level was significantly different between TW and OW, some transcripts involved in IAA synthesis and signal transduction pathways were also significantly differentially expressed in the TW/NW and TW/OW comparison groups. The expression of *ALDH1* (evm.model.group5.1511), which encodes the enzyme that catalyzes the conversion of indole-3-acetaldehyde to IAA, was upregulated in TW compared with NW and OW (Fig. 6). *UGT1* (evm.model.scaffold36.20), whose protein product catalyzes the production of the IAA precursor indolylmethyl-desulfoglucosinolate, was upregulated in TW compared with OW. *IAA1* (TCONS_00040006) was upregulated in TW compared with both NW and OW, *ARF5* (evm.model.group5.1332) was upregulated in TW compared with OW, and 4 *ARFs* were downregulated in TW compared with NW (Fig. 7).

### Gene ontology (GO) terms and KEGG pathway enrichment analyses of lncRNA target genes

The RNA-sequencing (RNA-seq) results showed that most lncRNAs were involved in cis regulation, followed sequentially by trans regulation and antisense regulation. The analysis of significantly enriched GO terms showed that the lncRNAs mainly regulated the expression of genes involved in nucleotide binding, cell wall and peripheral structure development (Table 1). The results of the KEGG pathway enrichment analysis of target genes (Additional file 4) showed that the cis-regulated target genes of these lncRNAs were enriched in the tryptophan metabolism and alpha-linolenic acid metabolism pathways, and the trans-regulated target genes were enriched in the phenylalanine metabolism, carotenoid biosynthesis and alpha-linolenic acid metabolism pathways. These metabolic pathways participate in plant hormone synthesis. The antisense-regulated target genes were most highly enriched in plant hormone signal transduction.

**Table 1:** Analysis of enriched GO terms for the lncRNA-targeted mRNAs

GO term	GO ID	Description	GeneRatio (2907)	BgRatio (15307)	P.adjust
Cis regulation					
Molecular Function	GO:0017076	purine nucleotide binding	507 (17.44%)	2188 (14.29%)	0.000025
	GO:0030554	adenyl nucleotide binding	456 (15.69%)	1946 (12.71%)	0.000025
	GO:0032555	purine ribonucleotide binding	496 (17.06%)	2139 (13.97%)	0.000025
	GO:0032553	ribonucleotide binding	502 (17.27%)	2172 (14.19%)	0.000025
	GO:0032559	adenyl ribonucleotide binding	446 (15.34%)	1906 (12.45%)	0.000027
	GO:0005515	protein binding	703 (24.18%)	3169 (20.7%)	0.000029
	GO:0043168	anion binding	556 (19.13%)	2469 (16.13%)	0.000102
	GO:0035639	purine ribonucleoside triphosphate binding	459 (15.79%)	2020 (13.2%)	0.000337
	GO:0000166	nucleotide binding	546 (18.78%)	2449 (16%)	0.000337
	GO:1901265	nucleoside phosphate binding	546 (18.78%)	2449 (16%)	0.000337
	GO:0005524	ATP binding	409 (14.07%)	1787 (11.67%)	0.000484
Cellular Component	GO:0005634	nucleus	160 (18.69%)	760 (14.36%)	0.021341
Trans regulation					
Cellular Component	GO:0005618	cell wall	15 (6.47%)	104 (1.97%)	0.006155
	GO:0071944	cell periphery	21 (9.05%)	193 (3.65%)	0.006695
	GO:0030312	external encapsulating structure	15 (6.47%)	115 (2.17%)	0.006695
Biological Process	GO:0055114	oxidation-reduction process	95 (15.97%)	1187 (10.36%)	0.005702

We explored the lncRNAs that regulated mRNAs in the phytohormone synthesis and signal transduction pathways, and a detailed description of this information is provided in Additional file 5. Interestingly, 165 lncRNAs were involved in regulating the expression of genes in the plant hormone signal transduction pathway. In this pathway, 5.26, 5.72 and 9.62% of the lncRNAs, exhibited cis regulation, trans regulation and antisense regulation, respectively (Table 2).

**Table 2:** Number of mRNAs regulated by lncRNAs in the hormone biosynthesis and signal transduction pathways

Pathway	Description	Pathway ID	The pattern of lncRNA regulation		
			Cis	Trans	Antisense
Carotenoid biosynthesis	ABA biosynthesis	ko00906	16 (0.75%)	9 (1.39%)	2 (1.28%)
Brassinosteroid biosynthesis	BR biosynthesis	ko00905	5 (0.23%)	3 (0.46%)	1 (0.64%)
Cysteine and methionine metabolism	ETH biosynthesis	ko00270	29 (1.35%)	10 (1.55%)	3 (1.92%)
Diterpenoid biosynthesis	GA biosynthesis	ko00904	12 (0.56%)	5 (0.77%)	0
Tryptophan metabolism	IAA biosynthesis	ko00380	19 (0.88%)	6 (0.93%)	0
alpha-Linolenic acid metabolism	JA biosynthesis	ko00592	24 (1.12%)	10 (1.55%)	4 (2.56%)
Phenylalanine metabolism	SA biosynthesis	ko00360	16 (0.75%)	8 (1.24%)	3 (1.92%)
Zeatin biosynthesis	ZT biosynthesis	ko00908	12 (0.56%)	5 (0.77%)	1 (0.64%)
Plant hormone signal transduction	Hormone signal transduction	ko04075	113 (5.26%)	37 (5.72%)	15 (9.62%)

### Regulation of lncRNAs and transcription factors in the plant hormone response during TW formation

**Fig. 8:**
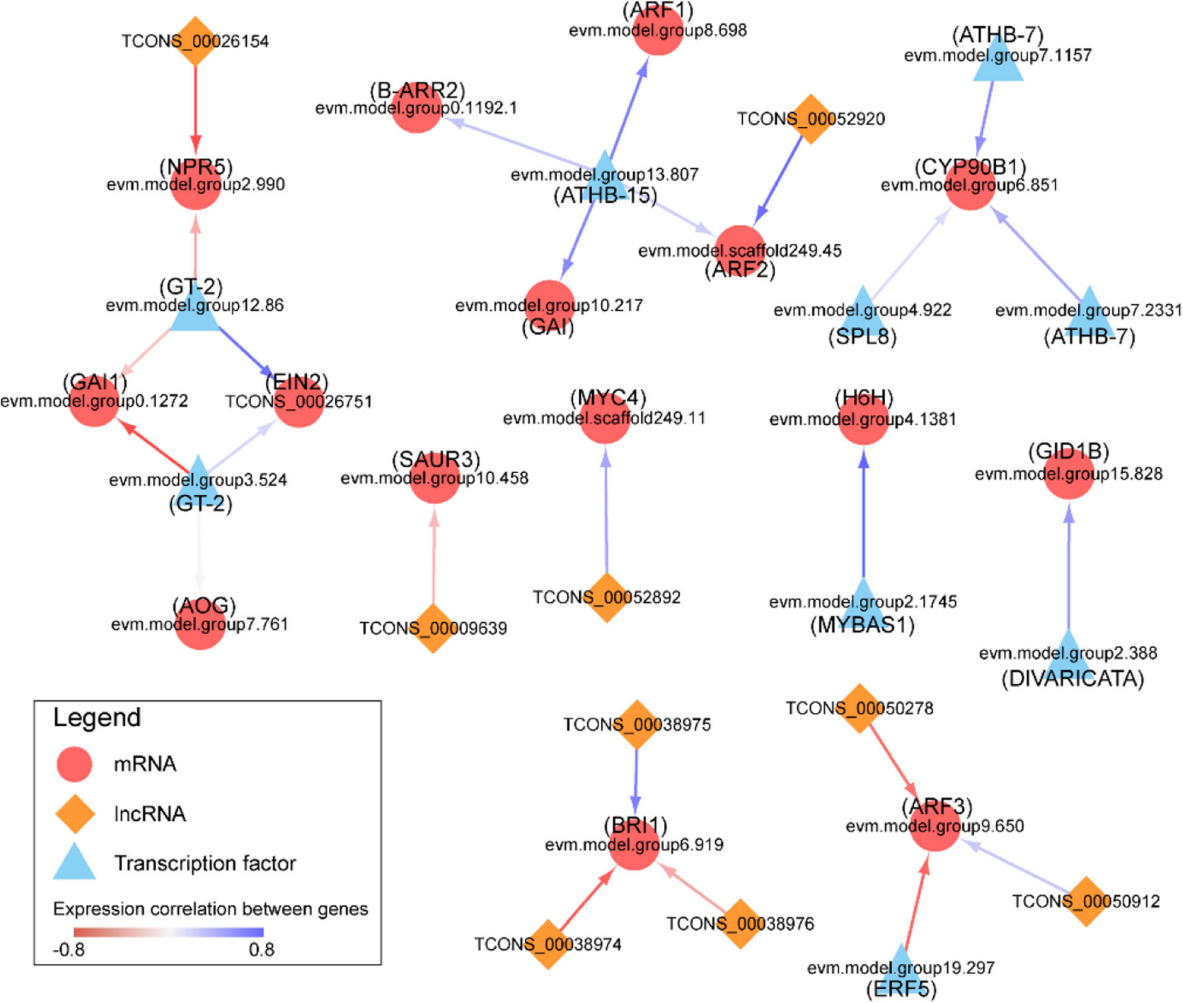
Regulatory network of lncRNAs, mRNAs and transcription factors

Six differentially expressed mRNAs involved in plant hormone biosynthesis and signal transduction were potentially regulated by cis-regulatory lncRNAs (Fig. 8). Because cis regulation requires the co-expression of lncRNAs and mRNAs, we further analyzed the correlations between the expression of these lncRNAs and mRNAs. This analysis revealed a strong correlation between *ARF2* (evm.model.scaffold249.45) expression and TCONS_00052920 expression (0.75). In addition, potential correlations between cis regulatory factors were observed between the BR signaling receptor gene *BRI1* (evm.model.group6.919) and three novel lncRNAs (TCONS_00038974, TCONS_00038975, and TCONS_00038976), but only TCONS_00038975 exhibited a strong positive correlation with evm.model.group6.919 (0.65). This pattern implied that TCONS_00052920 and TCONS_00038975 may be novel lncRNAs regulatingTW formation in *C. bungei*.

In addition, we explored the differentially expressed transcription factors (DETFs) that regulate these DEGs to construct a regulatory network (Additional file 6, Fig. 8). Most DETFs were positively correlated with the DEGs. The transcription factor ATHB-15 (evm.model.group13.807) regulated four target genes and was strongly positively correlated with two of them, *ARF1* (evm.model.group8.698) and *GAI* (evm.model.group10.217). Moreover, the *CYP90B1* (evm.model.group6.851) gene exhibited a potential regulatory relationship with three transcription factors, two of which belong to the HD-ZIP transcription factor family.

### qRT-PCR analysis

**Fig. 9:**
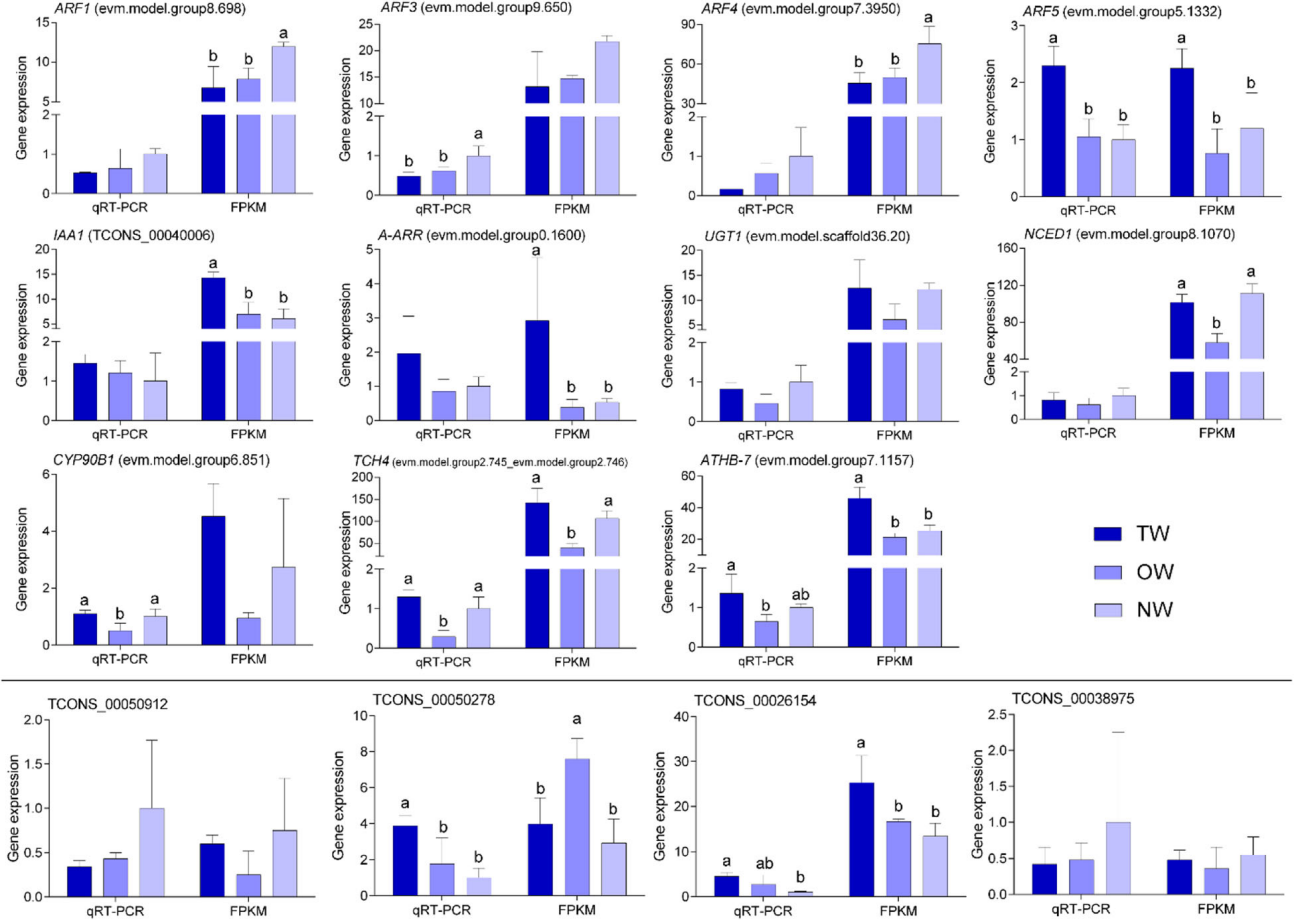
qRT-PCR validation of key genes in different wood types. The mRNA expression results are shown above the black line; the lncRNA expression results are shown below the black line. The different lowercase letters indicate significant differences at a significance level of 5%.

The expression levels of selected genes involved in plant hormone biosynthesis and signal transduction were measured using qRT-PCR to validate the results of the DEGs (Fig. 9). qRT-PCR results were consistent with the RNA-seq data, indicating that these genes were precisely the key players in regulating the phytohormone content, phytohormone response and induction of TW formation in *C. bungei*. In addition, the results of the quantitative fluorescence analysis of lncRNAs showed that the relative expression of only the TCONS_00050278 gene was different from the result obtained using RNA-seq, while the expression patterns of the other three lncRNAs in the three wood types were relatively consistent with the sequencing results.

### Phytohormones mediated TW formation in *C. bungei*

**Fig. 10:**
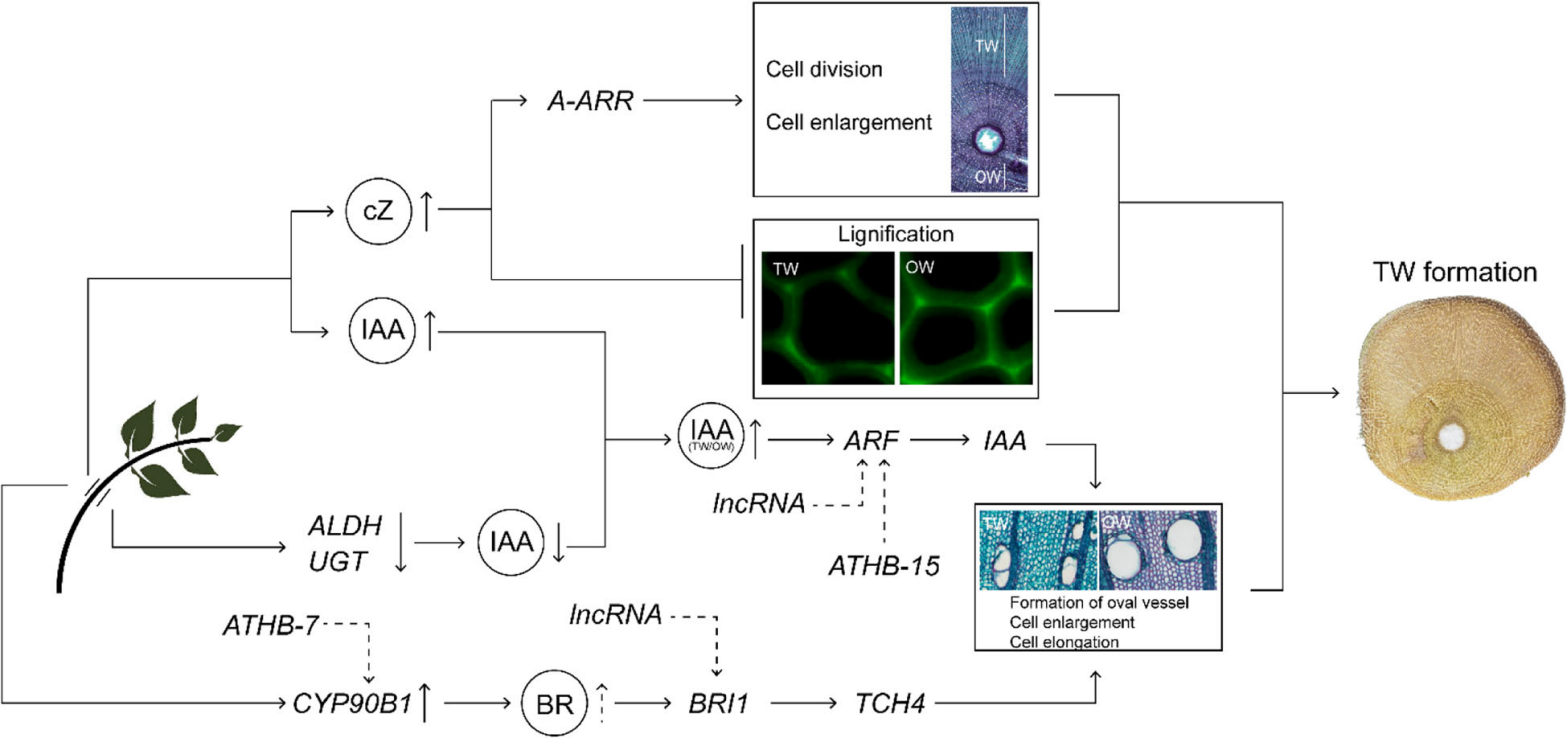
The proposed model of the mechanism by which multiple phytohormones regulate TW formation in *C. bungei*. The dotted line indicates the predicted result

Based on the results described above, we constructed a multiple hormone-mediated regulatory network of TW formation in *C. bungei* (Fig. 10). Mechanical stimulation induced the expression of genes involved in the IAA and BR biosynthesis pathways to alter the phytohormone content. In addition, the balance of IAA contents between the upper and lower stem was disrupted. It induced the expression of ATHB transcription factors and lncRNAs to regulate plant hormone signal transduction and activate their target genes (*A-ARR*, *ARF* and *TCH4*), and modulate the chemical and physical properties of fibers. At the same time, these changes altered the differentiation pattern of vessels and the cell division ability of cambium to decrease the lignification process in the stress area.

## Discussion

In our study, the vessels were smaller, and the vessel length:width ratio was significantly larger in TW than in OW and NW. Previous studies also confirmed that TW has smaller and longer vessels [10, 14]. In addition, the TW of *C. bungei* had a thinner secondary wall without a G-layer. This result is consistent with the absence of the G and S3 layers and the thinner cell walls observed in two Magnoliaceae species [31]. Moreover, the TW exhibited lower lignification than OW and NW according to the Raman spectra and microscopy images. According to earlier reports, both the results of the spectroscopic analysis and chemical analysis indicate a lower lignin content in TW than in OW or NW [31–33]. Fibers resulting from low lignification may be a prerequisite for the increase in of the growth stress [33].

We detected a higher IAA content in TW than in OW. We speculate that the synthesis of IAA in OW maybe significantly reduced, or that more IAA is transported to TW. This result was similar to the findings reported by Hellgren et al. (2004), who indicated that the endogenous IAA content did not differ between NW and TW in *P. tremula*, although OW contained less IAA than TW according to LC-MS data [23]. The cZ content in TW was also significantly higher than in OW and NW. As a cytokinin, cZ regulates cell proliferation. Cytokinins regulate vascular tissue differentiation and cell wall lignification [34–37]. The increased cZ content in TW may explain why the increase in the cell layers in the upper stem triggers eccentric growth and decreases lignification. Cytokinins usually cooperate with auxin to regulate xylem development [38]. According our results, significantly higher levels of both IAA and cZ were detected in TW than in OW, and these hormones may coordinately control TW formation. Furthermore, OW contained the highest GA1 level, while TW contained the highest GA7 level. According to reports, most GAs do not have biological activity but GA1, GA3, GA4 and GA7 are the major bioactive GAs, [39] and they may participate in TW formation. The effect of gibberellin on TW formation has also been reported. Jiang et al. (2008) wherein GA3 and GA4 were exogenously applied to seedling of *Fraxinus mandshurica*, which resulted in negative geotropism and promoted G-layer formation in the fiber cell lumen [40]. According to Wang et al. (2017), gibberellin signaling induces RGA protein degradation, thus activating *PtFLA6* gene expression and promoting TW formation in poplar [20]. Collectively, these results indicate that gibberellin is a positive factor regulating TW formation. However, our results did not indicate whether gibberellin was the key hormone regulating TW formation or its effects on TW formation in *C. bungei*, because the level of bioactive gibberellin varied among the wood types.

The *A-ARR* gene *PtRR7* is usually highly expressed in the vascular cambium in poplar [37]. FBR12 negatively regulates the expression of *AtAHP6*, *AtARR15* and *AtARR16* by forming a complex with CRE1 and AHP1 to inhibit xylem formation in *Arabidopsis thaliana* [41]. In the present study, the expression of *A-ARR* and *CRE*, *AHP* and *B-ARR* genes was upregulated and downregulated in TW, respectively. Based on the results of the studies described above, we speculated that a cytokinin (cZ) may activate the *A-ARR* gene in *C. bungei* and negatively regulate the expression of *B-ARR* gene to modulate cxylem development in TW. The *BRI1* and *TCH4* genes related to BR signaling were significantly differentially expressed in TW compared with NW or OW. Additionally, the *CYP90B1*, which catalyzes the synthesis of BR, was significantly upregulated in TW compared with OW. Thus, BR signaling may regulate TW formation. *PtCYP85A3*, which is the gene encoding a key rate-limiting enzyme in BR synthesis, is overexpressed in poplar and increases the height and radial growth without changing the cell wall thickness [42]. This finding confirms that BR is related to plant stem cell division and elongation. Recent reports have also shown that an exogenous BR treatment increases the xylem area, alters carbohydrate deposition, and increases the expression of cell wall differentiation-associated genes to promote TW formation in poplar [43–45]. Interestingly, TCH4 responds to mechanical stimulation and is regulated by steroids, and its expression is significantly associated with cell enlargement [46]. Xyloglucan endotransglycosylases (XETs) encoded by *TCH4* modify lignin polymers to alter the properties of the cell wall, such asextensibility and tensile strength [47]. In the present study, the activation of TCH4 in the TW of *C. bungei* may have changed the physical properties of the fiber cell wall. This relationship may be one of the key links in the mechanism underlying TW formation. Unfortunately, endogenous BR levels were not determined in this experiment.

The IAA content and the expression of genes involved in IAA biosynthesis and signal transduction were significantly different between TW and NW or OW in *C. bungei*. For instance, the *ALDH1*, *UGT1*, *IAA1* and *ARF5* genes were all upregulated in TW compared with NW or OW. Four *ARFs* were downregulated in TW compared with NW. As shown in the study by Yu et al. (2017), exogenous IAA increased the expression of the IAA transport-related genes *PIN1*, *ABCB1*, and *AUX2*, accelerating the polar transport of auxin and inducing TW formation [22]. Hellgren et al. (2004) did not observe significant differences int the endogenous IAA concentration in the cambium between TW and NW; thus, IAA was considered to have no correlation with TW formation [23]. However, Gerttula et al. (2015) found that the expression of the PIN3 at a specific location changed auxin polar transport, which was one of the mechanisms underlying TW formation, and this mechanism was regulated by gibberellin signaling [28]. *ARF5* plays an important role in the early stage of vascular formation in *A. thaliana* and participates in multiple developmental processes, including vascular development in leaves and embryos, by directly activating the expression of *ATHB8* and *PIN1* [48–50]. Moreover, auxin can regulate the expression of the BR receptor gene, and BR utilizes the *ARF7* and *IAA19* to regulate this downstream process [51, 52]. Notably, a significantly higher IAA level was detected in TW than in OW, and at the same time, the genes involved in the responses to IAA and BR signaling were also significantly differentially expressed during TW formation. Thus, IAA and BR might coordinate to control TW formation in *C. bungei*.

Gene expression is often regulated by intrinsic molecules. The mutation and regulation of lncRNAs play a key role in the expression of functional genes [53]. Here, we performed a preliminary analysis of the relationship between the expression of some lncRNAs and key genes. In our study, some *DEGs* (evm.model.scaffold249.45, evm.model.group6.919) involved in plant hormone biosynthesis and signal transduction were potential targets of lncRNAs (TCONS_00052920 and TCONS_00038975) through cis-regulation, according to the prediction of targeted relationships and the coexpression analysis. In addition, the transcription factor ATHB-15 and other HD-ZIP transcription factor family members were positively correlated with the DEGs related to IAA signal transduction. HD-ZIP transcription factors regulate several genes in the auxin signaling pathway, including the *ARF* gene, to regulate plant embryonic development [54]. Moreover, the auxin response factors BDL and MP/ARF5 are coexpressed with HD-ZIP transcription factors during vascular development in *A. thaliana* embryos [55]. Thus, in our study, the changes in the expression of the ATHB-15 transcription factor in the HD-ZIP family may be dependent on the participation of auxin in vascular development of TW.

## Conclusion

In this study, various plant hormones participated in *C. bungei* TW formation. Our results revealed a multiple hormone-mediated network regulating TW formation in *C. bungei*. TW exhibits significantly higher IAA and cZ contents than OW and NW; these plant hormone signals induced the expression of genes involved in IAA, cZ and BR signal transduction pathways. At the same time, some lncRNAs and transcription factors regulate the expression of some of the genes that were activated. Finally, these genes modulate the development of vessels and fibers in TW. This result provides a new basis for explaining the mechanisms of phytohormone-mediated TW formation.

## Methods

### Plant material

The tested *C. bungei* plants were grown at the experimental forest base at the Biandanzhao village (Luoyang, Henan, China; 112°33′E, 34°43′N). The *C. bungei* clone 8402 was grafted at the end of April 2017, and plants were bent at a consistent angle of 45° for 3 months to induce TW formation. The samples were taken from a height of 60 cm after bark removal. Samples from control plants (plants that were not subjected to bending) were collected at the same height. Information about the *C. bungei* specimens is available on the National Museum of Natural History website: http://coldb.mnhn.fr/catalognumber/mnhn/p/p03532196. The deposition number is P03532196.

**Fig. 11:**
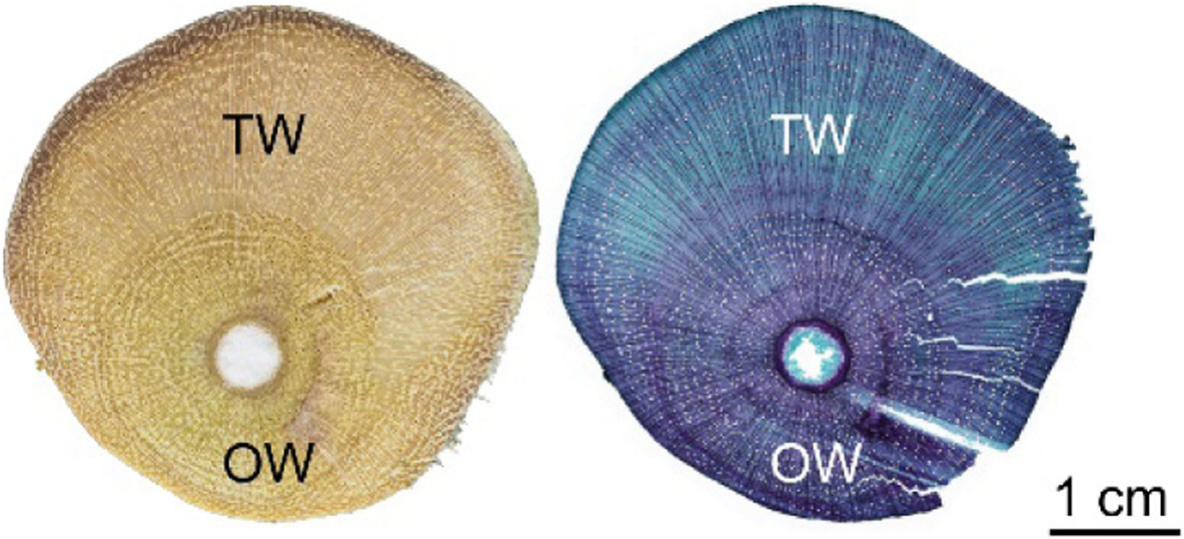
Collection of samples of different wood types

The parts that grew away from the center of the circle and stained blue-green with fast green were TW. The opposite properties were observed for OW (Fig. 11.). The tissues of each sample were divided according to the staining results of the sections. Five different individual plant samples were combined, and biological triplicates were used in subsequent experiments.

### Anatomy of xylem

Leica SM2010 R slicing microtome was used to produce the 30-μm xylem slices, which were incubated with 1% safranine dye for 12 h and dehydrated in a series of 30, 50, 75, and 95% ethanol and anhydrous ethanol solutions. The slices were then incubated with 0.1% fast green dye for 4 min and sealed with Canadian resin. The sizes of the fibers and vessels (fiber cell wall thickness and vessel length and width) in the xylem of TW, OW and NW were measured in 3 individual plants. One hundred fifty cells from 5 microscopic fields were randomly counted from TW, OW and NW in each individual plant, and 450 cells were evaluated for each wood type.

### Confocal Raman microscopy and Raman mapping

Tissue sections of the stem were imaged with a LabRam Xplora confocal Raman microscope (Horiba Jobin–Yvon, Paris, France) and an Olympus confocal microscope (Olympus, Tokyo, Japan) equipped with a linearly polarized 532 nm laser (Ventus VIS 532, Laser Quantum. A detailed description of the test procedures is provided in a previous study [56].

### LC-MS analysis of plant hormones

(1) Sample preparation and extraction: Fresh xylem samples were harvested, weighed, immediately frozen in liquid nitrogen, and stored at − 80 °C until further use. Plant materials (120 mg fresh weight) were frozen in liquid nitrogen, ground into powder, and extracted with methanol:water (8:2) at 4 °C. The extract was centrifuged at 12000×g at 4 °C for 15 min. The supernatant was collected and was evaporated to dryness under a nitrogen gas stream and reconstituted in methanol: water (3:7). The solution was centrifuged, and the supernatant was collected for the LC-MS analysis.

(2) High-performance liquid chromatography (HPLC): Extracts of the xylem samples were analyzed using an LC-ESI-MS/MS system (HPLC, Shim-pack UFLC SHIMADZU CBM30A system, www.shimadzu.com.cn/; MS, Applied Biosystems 6500 Triple Quadrupole, www.appliedbiosystems.com.cn/). The analysis conditions were as follows: HPLC column, Waters ACQUITY UPLC HSS T3 C18 (1.8 μm, 2.1 mm × 100 mm); solvent system, water (0.04% acetic acid):acetonitrile (0.04% acetic acid); gradient program, 95:5 V:V at 0 min, 5:95 V:V at 11.0 min, 5:95 V:V at 12.0 min, 95:5 V:V at 12.1 min, and 95:5 V:V at 15.0 min; flow rate, 0.35 mL/min; temperature, 40 °C; and injection volume: 5 μL. The effluent was alternatively connected to an electrospray ionization (ESI)-triple quadrupole linear ion trap (Q TRAP) mass spectrometer.

(3) ESI-Q TRAP-MS/MS: An API 6500 Q TRAP LC-MS/MS system equipped with an ESI Turbo Ion Spray interface, operating in positive ion mode and controlled by Analyst 1.6 software (AB Sciex) was used. The ESI source operation parameters were as follows: ion source, turbo spray; source temperature, 500 °C; ion spray (IS) voltage, 5500 V; curtain gas (CUR), 35.0 psi; and collision gas (CAD), medium. The declustering potential (DP) and collision energy (CE) of individual multiple reaction monitoring (MRM) transitions were calculated, with further DP and CE optimization. A specific set of MRM transitions was monitored for each period according to the plant hormones eluted within this period.

### Illumina high-throughput sequencing of mRNAs and lncRNAs

Total RNA was extracted from the TW, OW and NW using an RNA reagent kit (DP441; Tiangen Biotech, Beijing, China) according to the manufacturer’s protocol. Nine transcriptome libraries were constructed for 150-bp paired-end Illumina high-throughput sequencing of mRNAs and lncRNAs using the Illumina HiSeq™ 4000 platform (Illumina, USA) by Gene Denovo Biotechnology Co. (Guangzhou, China). The post sequencing data filtering and analysis methodology have been described in a previous study [57]. Cufflinks and Cuffcompare software [58, 59] were used to assemble transcripts and compare sequences to known sequences in the *C. bungei* genome (unpublished). The fragments per kilobase of transcript per million mapped reads (FPKM) value was used as an indicator to evaluate gene expression [59]. In the present study, transcripts with a fold change of ≥1.5 or ≤ − 1.5 and a *P* value of < 0.05 between various comparison groups were considered significant DEGs.

### Identification of lncRNAs

Three network databases were used to predict the protein-coding ability of the transcripts: CPC (http://www.mybiosoftware.com/cpc-0-9r2-assess-protein-coding%2D%2Dpotential-transcripts.html) (Kong et al. 2007), txCdspredict (http://hgdownload.soe.ucsc.edu/admin/jksrc.zip) [60], and CNCI (https://github.com/www-bioinfo-org/CNCI) [61]. In addition, lncRNAs were distinguished from mRNAs according to the thresholds. In the CPC and CNCI databases, the score thresholds were ≥ 0 for mRNA and < 0 for lncRNAs, and in txCdspredict, the thresholds were ≥ 500 for mRNAs and < 500 for lncRNAs. In addition, the Pfam database was used to further distinguish the mRNAs and lncRNAs, transcripts that were aligned to this protein database were predicted to be mRNAs, and transcripts that were not aligned were considered lncRNAs [62]. Transcripts that satisfied at least three predicted results were identified as lncRNAs.

### Regulatory relations between lncRNAs and mRNAs

Notably, lncRNAs usually regulate target genes in three ways: proximal regulation, which is termed cis-acting regulation; remote regulation, which is termed trans-acting regulation; and antisense regulation. The lncRNAs located upstream or within 20 kb downstream of an mRNA were identified as cis-acting lncRNAs. We analyzed the binding energy of the lncRNAs and mRNAs using RNAplex [63] software. The lncRNAs with a binding energy of < 30 and a distance of greater than 20 kb from their target mRNA were identified as trans-acting lnRNAs. Pearson’s correlation coefficients were calculated to evaluate coexpression relationships between lncRNAs and mRNAs. The software RNAplex (http://www.tbi.univie.ac.at/RNA/RNAplex.1.html) was used to predict the complementary correlations of antisense lncRNAs with mRNAs. The program includes the ViennaRNA package, and the prediction of the best base pairing was based on the calculation of the minimum free energy from the thermodynamic structure.

### qRT-PCR

The total RNA used for qRT-PCR was the same as the sample used for RNA-seq. qRT-PCR was performed in a 7500 Real-Time PCR System (Applied Biosystems, CA, USA) using TB Green Premix Ex Taq™ (TaKaRa, Dalian, China) according to the manufacturer’s instructions. The 2^–ΔΔCt^ method was used to calculate the relative expression levels [64]. The actin gene of *C. bungei* served as the reference gene, and detailed primer information is shown in Additional file 7.

### Statistical analysis and graphical presentation

The R program was used to measure significance with ANOVA and Duncan’s multiple comparisons tests. GraphPad 5 and Adobe Illustrator CS6 software were used to generate the figures. MultiExperiment Viewer software was used to construct the heat map of gene expression. The regulatory network was constructed using Cytoscape software.

## Supplementary information

**Additional file 1: Table S1.** The wood properties of different clones of *C. bungei.*

**Additional file 2: Table S2** Number of different types of lncRNAs.

**Additional file 3: Table S3** The annotation of DEGs involved hormone biosynthesis and signal transduction.

**Additional file 4: Figure S1** KEGG pathway enrichment analysis of lncRNA target genes. (A) cis regulation, (B) trans regulation, (C) antisense regulation.

**Additional file 5: Table S4** The annotation of genes regulated by potential lncRNAs through cis regulation. **Table S5** The annotation of genes regulated by potential lncRNAs via trans regulation. **Table S6** The annotation of genes regulated by potential lncRNAs via antisense regulation.

**Additional file 6: Table S7** The annotation of genes regulated by lncRNAs and transcription factors in the regulatory network. **Table S8** The correlations between the expression of genes and regulators.

**Additional file 7: Table S9** Primer information.

## Data Availability

The transcriptome sequence data have been deposited to the NCBI via Sequence Read Archive (SRA) with the accession numbers: PRJNA559964.

## Abbreviations

TW: Tension wood
OW: Opposite wood
NW: Normal wood
LC-MS: Liquid chromatography-mass spectrometry
lncRNA: Long noncoding RNA
G-layer: Gelatinous layer
KEGG: Kyoto Encyclopedia of Genes and Genomes
DEGs: Differentially expressed genes
FPKM: Fragments per kilobase of transcript per million mapped reads
