# Supplementary figures and images for: Genome-wide analysis of lncRNA and mRNA expression and endogenous hormone regulation during tension wood formation in Catalpa bungei
Source: BMC Genomics. 2020 Sep 5;21:609. doi: 10.1186/s12864-020-07044-5 (PMC7487903; doi:10.1186/s12864-020-07044-5)

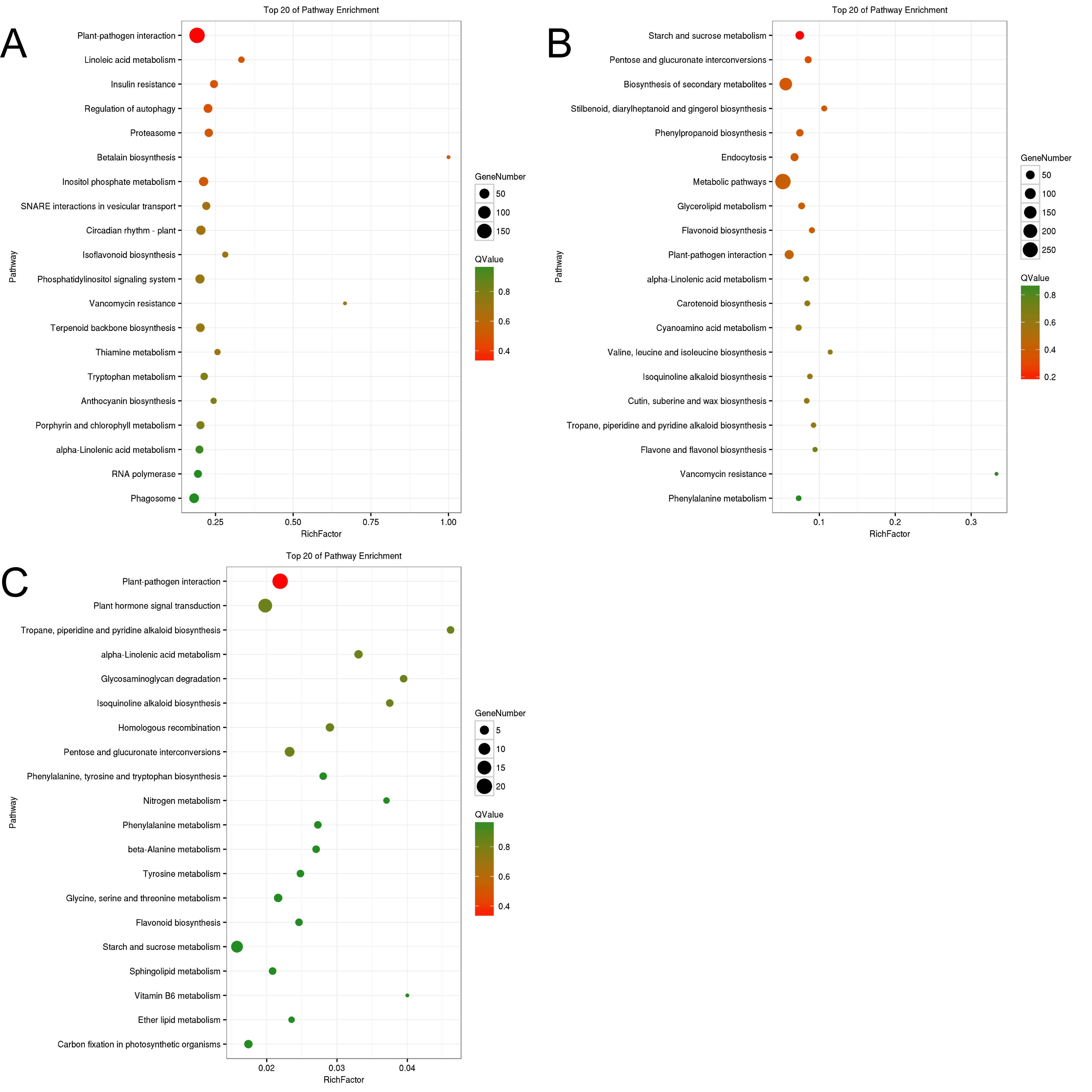

Supplement: Supplementary file 4 — Additional file 4: Figure S1 KEGG pathway enrichment analysis of lncRNA target genes. (A) cis regulation, (B) trans regulation, (C) antisense regulation. [file 12864_2020_7044_MOESM4_ESM.tif]
